# OxyR is involved in coordinate regulation of expression of *fimA* and *sod* genes in *Porphyromonas gingivalis*

**DOI:** 10.1111/j.1574-6968.2008.01116.x

**Published:** 2008-03-18

**Authors:** Jie Wu, Xinghua Lin, Hua Xie, Robert Burne

**Affiliations:** School of Dentistry, Meharry Medical College Nashville, TN, USA

**Keywords:** fimbriae, SOD, OxyR

## Abstract

Survival of *Porphyromonas gingivalis* in the constantly changing oral environment depends on its ability to alter gene expression. We demonstrate here that *P. gingivalis* activates superoxide dismutase expression in response to oxidative stress and represses expression of FimA, a subunit of major fimbriae. Coordinated expression of *fimA* and *sod* is regulated by the redox-sensing transcription factor OxyR. Mutations in the *oxyR* gene result in a decreased expression of *sod* and in an elevated expression of *fimA*. In addition, we provide evidence that regulation of expression of *fimA* and *sod* by OxyR is mediated by direct interaction of OxyR and the promoters of these two genes. These results suggest that OxyR plays an important role in regulation of expression of virulence genes in *P. gingivalis*.

## Introduction

*Porphyromonas gingivalis* is a secondary colonizer of dental plaque, and is significantly more prevalent in both supra- and subgingival plaque samples from periodontitis subjects in comparison with samples from healthy subjects (Ximenez-Fyvie *et al.*, 2000a). The organism is associated with several forms of periodontitis (Socransky *et al.*, 2002). It has been well recognized that expression of *fimA*, encoding a subunit (FimA) of long (major) fimbriae, is required for colonization of *P. gingivalis* on a variety of oral surfaces (Lamont & Jenkinson, 1998). A *P. gingivalis* strain with *fimA* deficiency has a diminished capacity to adhere to human gingival fibroblasts and epithelial cells (Hamada *et al.*, 1994). FimA also participates in coaggregation with various early plaque-forming bacteria, such as *Actinomyces viscosus*, *Streptococcus gordonii*, *Streptococcus oralis* and *Treponema denticola* (Goulbourne & Ellen, 1991; Lamont *et al.*, 1993; Amano *et al.*, 1997). Expression of the *fimA* gene is modulated in *P. gingivalis* in response to environmental cues, such as temperature and ion supply (Amano *et al.*, 1994; Xie *et al.*, 1997). We have reported earlier that *fimA* expression is repressed in the presence of another oral bacterium, *Streptococcus cristatus* (Xie *et al.*, 2000). A two-component system (*fimS/fimR*), first reported by Hayashi *et al.* (2000), is found to be involved in the fimbriation of *P. gingivalis*. Sequence analysis demonstrates that FimS/FimR contain conserved sequences that have been suggested to be phosphorylation sites for transmitters and receivers. Disruption of *fimS* or *fimR* results in significant reduction in FimA production. Recently, Nishikawa *et al.* (2004) reported that FimR controls the expression of several genes clustered around the *fimA* locus, and that the recombinant FimR does not bind to the *fimA* gene but binds to the first gene in the *fimA* cluster, *pg2130*.

Pathogenicity of *P. gingivalis* involves the products of many genes. One important virulence factor is superoxide dismutase (SOD), which is required for modest aerotolerance of *P. gingivalis*. A *sod*-deficient mutant is more sensitive to atmospheric oxygen (Lynch & Kuramitsu, 1999). It is reported that *P. gingivalis* can alter its expression of FimA and SOD under the conditions observed in an inflamed periodontal pocket, such as an elevated temperature (Amano *et al.*, 1994). Expression of the *fimA* gene is decreased in *P. gingivalis* grown at 39 °C, whereas expression of *sod* is increased, suggesting a possible coordinated regulation of *fimA* and *sod*. Recent studies have shown that expression of *sod* is positively regulated by a redox-sensing transcription activator, OxyR (Diaz *et al.*, 2006; Ohara *et al.*, 2006). It remains unknown whether *P. gingivalis fimA* is one of the OxyR regulons. We hypothesize that expression of *fimA* and *sod* may be coordinately regulated through OxyR. Here we report that *P. gingivalis* selectively up-regulates expression of *sod* and represses *fimA* expression under aerobic conditions, and that the *oxyR*-deficient mutant increases *fimA* expression and promotes biofilm formation.

## Materials and methods

### Bacterial strains and growth conditions

*Porphyromonas gingivalis* strains were grown from frozen stocks in Trypticase soy broth (TSB) or on TSB blood agar plates, supplemented with yeast extract (1 mg mL^−1^), hemin (5 μg mL^−1^), and menadione (1 μg mL^−1^), at 37 °C in an anaerobic chamber (85% N_2_, 10% H_2_, 5% CO_2_). *Escherichia coli* DH5α was used as the host for plasmids. *Escherichia coli* strains were grown in Luria–Bertani broth at 37 °C. Antibiotics were used when appropriate, at the following concentrations: gentamicin (100 μg mL^−1^), erythromycin (10 μg mL^−1^), ampicillin (50 μg mL^−1^), kanamycin (50 μg mL^−1^), and tetracycline (5 μg mL^−1^).

### Experimental oxidative stress conditions

*Porphyromonas gingivalis* 33 277 (2 × 10^6^) were spotted on TSB blood agar plates and grown anaerobically for 48 h. The cells were then divided into two groups. One group was continuously incubated in the anaerobic chamber for another 3, 6, or 24 h. The other group was exposed to air at 37 °C for the same time periods.

### RNA isolation and reverse transcription (RT)-PCR

*Porphyromonas gingivalis* strains were grown to late-exponential phase (OD_600 nm_, 1.0–1.2) in 5 mL of TSB. Bacteria were harvested by centrifugation at 10 000 ***g*** and mixed in Trizol Reagent (Invitrogen, Carlsbad, CA). The RNA in the supernatant was then purified using an RNeasy mini spin column (Qiagen, Valencia, CA). RNA samples were digested on the column with RNAse-free DNAse. The total RNA was tested using an Agilent 2100 Bioanalyzer to insure the quality of the samples. RT-PCR was conducted using SuperScript™ One-Step RT-PCR Systems (Invitrogen) with about 10 ng of total RNA as a template, and the conditions indicated by the supplier, using 26 PCR cycles. For each reaction, a negative control with *Taq* polymerase and without reverse transcriptase was included. The RT-PCR products were visualized by 1.2% agarose gel.

### Construction of the *oxyR* mutant

An insertional *oxyR* mutant was generated using ligation-independent cloning (LIC) of PCR mediated mutagenesis (LIC-PCR) (Aslanidis & de Jong, 1990; Xie *et al.*, 2007). A 2.1-kb *ermF*-*ermAM* cassette was introduced into the *oxyR* gene by three steps of PCR to yield a 3884-bp *oxyR-erm-oxyR* DNA fragment as described previously (Fletcher *et al.*, 1995). This 3884-bp fragment was then introduced into *P. gingivalis* 33 277 by electroporation (Wu *et al.*, 2007). The *oxyR* deficient mutants were constructed via a double crossover event that replaces the *oxyR* with the *oxyR-erm-oxyR* into the 33 277 chromosome. The mutants were selected on TSB plates containing erythromycin (5 μg mL^−1^). The insertional mutation was confirmed by PCR analysis, and the mutants were designated as *P. gingivalis* OxyRE.

### Production of OxyR recombinant protein

The DNA fragment encoding OxyR was amplified by PCR with primers rOxyR-F and rOxyR-R, which produced a 927-bp PCR product (Table 1). The PCR products were then cloned into pCRII-TOPO (Invitrogen, Carlsbad, CA). The Recombinant OxyR (rOxyR) was expressed in *E. coli* using a pET protein expression system (Novagen, Madison, WI). The DNA fragment of *oxyR* was subcloned into the pET-30b downstream of a histidine tag. The rOxyR was expressed in *E. coli* BL21 (DE3) cells carrying the pET-30b/OxyR plasmid in the presence of isopropyl-β-d-thiogalactopyranoside and kanamycin. His-tagged rOxyR was purified with Ni^2+^-charged His-bind resin (Novagen, Madison, WI). The His-tag on the recombinant protein was cleaved with enterokinase and removed by His-bind resin. Enterokinase was then removed using Ekapture agarose. The rOxyR was used to generate rabbit anti-OxyR antibody (Lampire, Ottsville, PA).

**Table 1:** Oligonucleotide primers used in this study

Primer name	Primer sequences (5′–3′)	Applications
fimA-F	CGGAACGAATAACCCAGAGA	For RT-PCR of *fimA*
fimA-R	CTGACCAACGAGAACCCACT	
sod-F	AATTCCACCACGGTAAGCAC	For RT-PCR of *sod*
sod-R	GAGCCGAATTGTTTGTCGAT	
oxyRF	TCGCAAGCCAAGCAAATAC	For RT-PCR of *oxyR*
oxyRR	GACACGAGGCAGGAGATAGG	
pg1737F	ATGAATCCGATCCGCCACCAC	For RT-PCR of *pg1737*
Pg1737R	GCCTCCCATCCCAAAGCACT	
pg0270(1)F	GGGCTGCGATGAAGAAAGAT	For creating the *oxyR* mutant
pg0270(2)R	GATTCTTCCGCGCATACACT	
pg0270(1)F2	TCGAGAAGAGAATCCTGATGTG	
pg0270(2)R2	TCCGAACAGCAAAAGAACAG	
pg0270(1)R-erm	GATGTTGCAAATACCGATGAGCATTCGAGCTGCTGTATATTC	
pg0270(2)F-erm	CCTCTAGAGTCGACCTGCAGACAGGGCAGCATTTGGCTTG	
erm-F	GCTCATCGGTATTTGCAACA	
erm-R	CTGCAGGTCGACTCTAGAGG	
rOxyR-F	ATGAATATACAGCAGCTCG	For production of OxyR recombinant protein
rOxyR-R	TCAAGCCAAATGCTGCCCT	
fimAProm-F	CGACGCTATATGCAAGACAA	For generating biotin-labeled *fimA* promoter region
fimAProm-R	Bio-TGTAACGGGTTCTGCCTCGT	
fimAProm-RC	TGTAACGGGTTCTGCCTCGT	For generating cold *fimA* promoter region
sodProm-F	Bio-CTTTTCGCCCGAAGGTTTTATC	For generating biotin-labeled sod promoter region
sodProm-R	AACGTCTGATTTTTATTGTAATTAAG	
sodProm-FC	CTTTTCGCCCGAAGGTTTTATC	For generating cold *sod* promoter region
mfaProm-F	CTCTCGCGAGGGTCAATATC	For generating biotin-labeled *sod* promoter region
mfaProm-R	Bio-CGTCTTACCGGCTTCCCTAT	
mfaProm-RC	CGTCTTACCGGCTTCCCTAT	For generating cold *sod* promoter region
16S rRNAF	TGTAGATGACTGATGGTGAAA	For real time PCR
16S rRNAR	ACTGTTAGCAACTACCGATGT	

### Chromatin immunoprecipitation (ChIP) qPCR assay

A ChIP assay was conducted following the description by (Nishikawa *et al.*, 2004). Briefly, formaldehyde was added to the *P. gingivalis* 33 277 culture (20 mL) to a final concentration of 1%. The crossing-link reaction was stopped by the addition of glycine (125 mM). Cells were resuspended in 2 mL of lysis buffer (20 mM Tris-HCl pH 8.0, 10 mM EDTA, 0.5 mg mL^−1^ TLCK, 10 mg mL^−1^ lysozyme) for 10 min at room temperature, followed by the addition of an equal volume of 2 × immunoprecipitation (IP) buffer (0.1 M Tris-HCl pH 7.0, 0.3 M NaCl, 2% Triton X-100, 0.2% sodium deoxycholate). Cells were sonicated to fragment chromosomal DNA which was used as the ‘input’ fraction for the ChIP assay.

Anti-OxyR antibody (25 μL mL^−1^) was added to the input fraction and rotated overnight at 4 °C, and complexes were then incubated for 1 h with 20 μL bed volume of pre-equilibrated protein A Sepharose CL-4B beads (Sigma). After washing, antibody–protein–DNA complexes were treated with 30 μL elution buffer (1% sodium dodecylsulphate, TE pH 8.0, 10 mM dithiothreitol) for 30 min at 37 °C. The eluates were used as the output fraction for real-time PCR analyses. Preimmune rabbit serum was used as a control. Input and output fractions were treated with RNAse A (10 μg mL^−1^), and proteinase K (2 μg mL^−1^, Sigma). DNA samples were precipitated by ethanol and resuspended in 30 μL dH_2_O.

Real-time PCR analysis of ChIP samples was performed using the QuantiTect SYBR Green PCR Kit (Qiagen) on the iCycler MyiQ™ Real-Time PCR detection system (Bio-Rad Laboratories Inc.) according to the manufacturer's instructions. Primers were designed to amplify promoter sequences of *fimA, sod*, and *pg1737*. Primers are listed in Table 1. Amplification reactions consisted of an initial activation at 95 °C for 15 min, and 40 cycles of 94 °C for 15 s, 58 °C for 30 s, and 72 °C for 30 s. Data were collected during the extension step and were expressed in arbitrary fluorescence units per cycle. A melting curve was used at the end to confirm that there was only one peak and only one product. Enrichment values (fold) were calculated according to output/input (Nishikawa *et al.*, 2004).

### Electrophoretic mobility shift assay (EMSA)

EMSAs were performed using the LightShift Chemiluminescent EMSA Kit (PIERCE, Rockford, IL) as described previously (Wu *et al.*, 2007). Biotin labeled DNA fragments were generated using 5′ biotin incorporated primers (Table 1). Binding of rOxyR to DNA was carried out in a 20-μL reaction mixture containing 20 fmol biotin-labeled DNA, 10 mM Tris, pH 7.5, 50 mM KCl, 1 mM dithiothreitol, 10 ng μL^−1^ poly (dI-dC), 2% glycerol, 0.05% NP-40, 2 mM MgCl with various amounts of purified rOxyR protein (0, 2.5, 5 and 10 μg) at room temperature for 30 min. Samples were then loaded and run into a 5% nondenaturing polyacrylamide gel in 0.5 × Tris-Borate-EDTA buffer. The DNA and protein complexes were then transferred to a positively charged nylon membrane (380 mA, 30 min). The biotin end-labeled DNA was detected using the Streptavidin-Horseradish Peroxidase Conjugate and the Chemiluminescent Substrate. Each EMSA experiment was repeated at least three times.

#### Biofilm assay

*Porphyromonas gingivalis* were grown overnight in TSB, and cells were then resuspended in 4 mL fresh media to an OD_600 nm_ of 0.1 in a six-well plate (COSTAR) coated with human whole saliva and grown for 14 h. After the unbound cells were removed, each well was washed with 4 mL phosphate-buffered saline (PBS) three times. The cells in the *P. gingivalis* biofilm were lysed with lysis solution (Solution A, Invitrogen). DNA was extracted using Easy-DNA kit (Invitrogen) and resuspended in 50 μL TE buffer. The cells in biofilms were enumerated using QuantiTect SYBR Green PCR Kit with *P. gingivalis* species-specific 16S rRNA gene primers (Tran & Rudney, 1999).

Standards used to determine *P. gingivalis* cell numbers were prepared using genomic DNAs from the wild-type strain 33 277. A fresh culture of *P. gingivalis* 33 277, grown in TSB, was serially diluted in PBS and plated on TSB plates to obtain CFU per milliliter at each dilution. DNA was also isolated from the dilutions and a qPCR assay was run, as described above, to determine cell number. Three trials were performed on three separate cultures.

## Results

### Coordinate expression of *fimA* and *sod* in *P. gingivalis* in response to oxidative stress

**Fig. 1:**
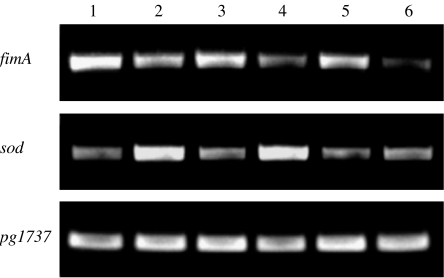
Coordinate expression of *fimA* and *sod* in *Porphyromonas gingivalis* in response to oxidative stress. *Porphyromonas gingivalis* 33 277 was grown on TSB plates for 48 h anaerobically. The bacteria cells were continuously incubated in the anaerobic chamber for another 3, 6, or 24 h (lanes 1, 3, 5) or exposed to air for 3, 6, or 24 h (lanes 2, 4, 6). Expression of *fimA* and *sod* was determined using RT-PCR. Glycosidase (*pg1737*) expression served as a control.

To determine whether expression of *fimA* and *sod* is coordinately regulated, we compared the mRNA level of these genes in response to oxidative stress using RT-PCR. As expected, expression of *sod* was increased in *P. gingivalis* 33 277 after the organism was exposed to air for 3 or 6 h (Fig. 1). An additional 24 h culture in an anaerobic chamber or in aerobic conditions had a notable affect on *sod* expression, although mRNA levels of *sod* were enhanced in *P. gingivalis* cells grown under oxidative stress, suggesting that expression of the *sod* is regulated by both oxidation stress and growth rate. In contrast to elevation of *sod* expression, expression of *fimA* was repressed when *P. gingivalis* was exposed to oxidative stress. In agreement with our earlier observation and in contrast to *sod*, expression of *fimA* was not affected by growth rate (Xie *et al.*, 1997). As a control, expression of *pg1737* encoding glycosidase was also examined, and no significant difference was detected in the mRNA levels when *P. gingivalis* was cultured either aerobically or anaerobically. The data demonstrate that expression of *fimA* and *sod* in *P. gingivalis* is coordinately regulated in response to oxidative stress.

### Role of OxyR in coordinate regulation of *sod* and *fimA*

**Fig. 2:**
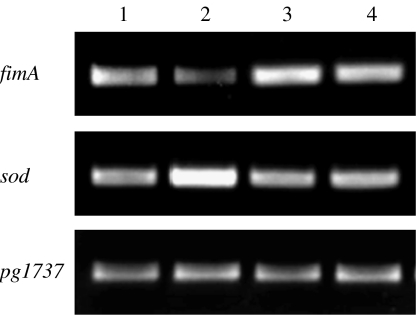
Expression of *fimA* and *sod* in wild-type strain 33 277 and in the *oxyR* mutant. Expression level of *fimA* and *sod* was determined by RT-PCR on RNA isolated from 33 277 grown anaerobically (lane 1), and aerobically (lane 2), or from the *oxyR* mutant grown anaerobically (lane 3), and aerobically (lane 4). Glycosidase (*pg1737*) expression served as a control.

Previous studies have shown that expression of *P. gingivalis sod* is regulated by redox-sensing transcription activator OxyR (Diaz *et al.*, 2006; Ohara *et al.*, 2006). We postulated that OxyR may be also involved in *fimA* expression in *P. gingivalis*. To test the hypothesis, we constructed an *oxyR* mutant, *P. gingivalis* oxyRE, and expression of *fimA* and *sod* in the mutant was compared with that in wild-type 33 277. Higher expression level of *fimA* was observed in the *oxyR* mutant compared with the level in 33 277 (Fig. 2), suggesting that OxyR acts as a repressor of *fimA* in *P. gingivalis*. Comparable expression levels of *fimA* and *sod* were observed when the *oxyR* mutant was grown aerobically vs. anaerobically, clearly demonstrating that the *oxyR* mutant lost the ability to respond to oxidative stress. In addition, mutations in *oxyR* did not affect expression levels of *sod* when the *P. gingivalis* was grown anaerobically, suggesting that *P. gingivalis* OxyR only functions in its oxidized form resembling *E. coli* OxyR (Mongkolsuk & Helmann, 2002).

**Fig. 3:**
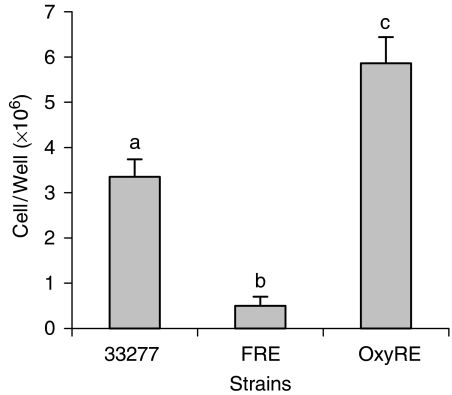
Effect of OxyR and FimR on formation of *Porphyromonas gingivalis* biofilms. The ability of biofilm formation of wild-type strain 33 277, the *fimR* mutant (FRE), and the *OxyR* mutant (OxyRE) by quantization of cells bound on saliva coated 6 well plates using qPCR. The measurements were performed in triplicate; mean values are shown. Means with different letters are significantly different (*P* < 0.01; two-way anova and Student–Newman–Keuls Test).

To determine if overexpressing FimA in the *oxyR* mutant has an impact on the binding ability of the strain, we compared biofilm formation among wild-type 33 277, the *fimR* mutant (FRE), and the *oxyR* mutant (OxyRE). We counted the number of bacteria in each biofilm using real-time PCR analysis. The number of bacteria in the FRE biofilms was sevenfold lower than in the wild-type strain. However, a twofold increase in bacteria was observed in biofilms of the *oxyR* mutant compared with wild-type 33 277 (Fig. 3). These findings demonstrate that OxyR influences the biofilm-forming potential of *P. gingivalis*.

### Binding of OxyR to the *fimA* promoter region

**Fig. 4:**
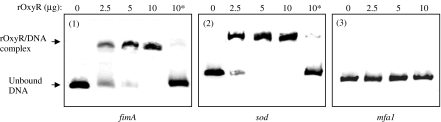
Interaction of rOxyR with the promoter region of *fimA* and *sod*. Electrophoretic mobility shift assays were performed in the presence or absence of rOxyR and 20 fmol biotin-labeled DNA. Increasing amounts of rOxyR were used in the assays. Asterisks indicate that 100-fold excess amounts of each specific competitor unlabeled probe were added to the reaction mixture with labeled probe.

One possible mechanism for regulation of *fimA* expression by OxyR is the direct interaction of OxyR with the *fimA* promoter. To assess if the interaction occurs, we performed electrophoretic mobility shift assay. The promoter regions of *fimA* (positioned from −22 to −190) (Xie & Lamont, 1999) and *sod* (positioned from −1 to −210) were generated by PCR with the 5′ biotin-labeled primers (Table 1). The promoter region (positioned from +18 to −138) of *mfa1*, a gene encoding component of the short fimbriae of *P. gingivalis*, was used as a control. Recombinant OxyR (rOxyR) was expressed in a pET Expression System and purified from *E. coli*. As shown in Fig. 4, the DNA fragments of the *sod* and the *fimA* promoter regions were shifted in the presence of the rOxyR. As the concentration of rOxyR increased, the retarded protein–DNA complex became more evident, with a parallel loss of uncomplexed *sod* promoter DNA. Our data also suggested that the DNA binding affinity to the *sod* promoter region was higher than that to the *fimA* promoter region, at least in the context of our incubation conditions. Complete retardation of *sod* promoter-rOxyR complex was detected with as little as 5 μg rOxyR, whereas 10 μg rOxyR was required for a complete retardation of the *fimA* promoter fragment (Fig. 4). This binding activity was blocked by addition of cold probe. There was no DNA shift detected when rOxyR was incubated with the promoter region of *mfa1*. These data show that OxyR protein can bind specifically to the *fimA* and the *sod* promoter regions *in vitro*, acting as an activator of *sod* and as a repressor of *fimA*.

**Fig. 5:**
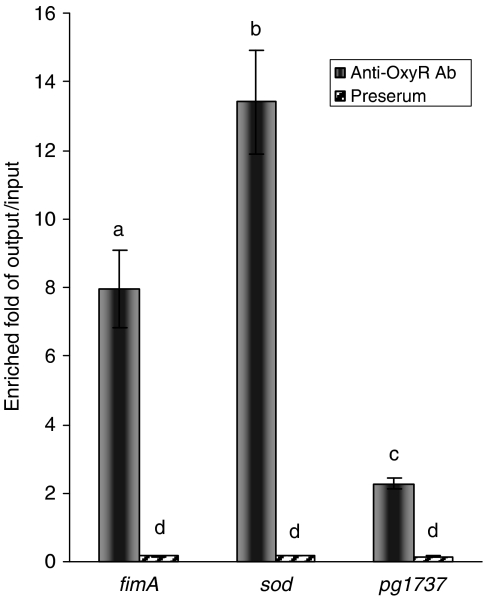
qPCR analysis of ChIP complex DNA. Immunoprecipitations were performed in the presence of anti-OxyR antibodies or preserum. The precipitated DNAs were amplified using promoter sequences of specific primers for *fimA*, *sod*, and *pg1737* (as a negative control). Means with different letters are significantly different (*P* < 0.01; two-way anova and Student–Newman–Keuls Test).

Using a ChIP qPCR assay, we further examined interactions between OxyR and the *fimA* promoter *in vivo*. *Porphyromonas gingivalis* 33 277 grown to late log phase was exposed to air for 3 h and then treated with formaldehyde to cross-link DNA-protein complexes. OxyR–DNA complexes were immunoprecipitated with anti-OxyR antibodies. A parallel sample using preimmune serum from the same rabbit was run as a background reference control. The immunoprecipitated DNA complexes were measured using real-time PCR analysis. As shown in Fig. 5, the promoter regions of *fimA* and *sod* were enriched eight- or 14-fold, respectively, by immunoprecipitation with anti-OxyR antibodies compared that to the twofold enrichment of the control gene, *pg1737*. Moreover, the enrichments of the promoter regions of *fimA* and *sod* were not observed in *P. gingivalis* 33 277 grown in aerobic conditions (data not shown). Although we could not rule out interaction of OxyR and the promoter region of *sod* in *P. gingivalis* grown under anaerobic conditions, it is likely that, at least, this interaction is enhanced when those bacteria were exposed to air. These results also indicate that OxyR binds to both *sod* and *fimA* promoter regions *in vivo*, although a relatively higher affinity is found for the DNA sequence of the *sod* promoter.

## Discussion

*Porphyromonas gingivalis* is a gram-negative and anaerobic bacterium commonly found in subgingival plaque, a causative agent of periodontitis (Ximenez-Fyvie *et al.*, 2000b). Although the deep periodontal pocket and bacterial biofilms create safety niches for *P. gingivalis*, the organism can be exposed to oxidative stress conditions in the oral cavity, especially in its early stages of colonization. One mechanism used by *P. gingivalis* to protect itself from oxidative damage is to increase expression of oxidative stress related-genes. The results from recent two-dimensional electrophoresis and real-time PCR analyses demonstrate an elevated expression of SOD and alkyl hydroperoxide reductase (AhpC) at both transcriptional and translational levels in *P. gingivalis* after exposure to oxidative stress (Diaz *et al.*, 2006; Ohara *et al.*, 2006). The ability to selectively produce enzymes under regulation by oxygen switching transcriptional factors allows *P. gingivalis* to survive under aerobic conditions. The results of the present studies confirm elevation in *sod* gene expression in *P. gingivalis* in response to oxygen levels. In addition, we demonstrate here that the expression of FimA, a major component of the long fimbriae, is also regulated in response to oxidative stress. Expression of *fimA* is repressed in *P. gingivalis* under aerobic conditions, which is presumably necessary to conserve its energy against oxidative stress. Up-regulating expression of oxidative stress-related genes such as *sod*, and repressing expression of other nonessential cellular components under the aerobic condition such as *fimA*, may be critical strategies for the bacteria's survival in the oral cavity and in periodontal pockets with increased superoxide levels produced from neutrophils.

We have identified the OxyR as a transcription factor that coordinately regulates target genes in response to oxidative stress. The *oxyR* is widely distributed in most Gram-negative and some Gram-positive bacteria (Mongkolsuk & Helmann, 2002). OxyR is considered as a global regulator, since the OxyR regulon of *E. coli* includes genes involved in peroxide metabolism and protection, redox balance, and some important regulators such as the ferric uptake regulator, Fur (Zheng *et al.*, 2001; Varghese *et al.*, 2007). The molecular basis for OxyR regulation has been well characterized in *E. coli*. OxyR is a tetrameric DNA-binding protein that is activated under oxidative stress by forming a disulphide bond between two Cys residues (Zheng *et al.*, 1998). Our genetic analysis of the *P. gingivalis oxyR* gene reveals that OxyR acts as a repressor of *fimA* in *P. gingivalis*, and that *fimA* is a member of the *oxyR* regulon. The mutation in *oxyR* results in an elevated expression of the *fimA* gene. The *oxyR* deficient mutants have lower expression levels of *sod*, which reduces the ability of the organism to adapt to oxidative stress. However, expression of *sod* in the wild-type strain and in the *oxyR* mutant is at similar levels when *P. gingivalis* cells were cultured in an anaerobic chamber. This finding suggests that, like *E. coli*, OxyR is activated under oxidative stress, and its activated form may have higher affinity to the promoter region of *sod* (Mongkolsuk & Helmann, 2002). Nevertheless, activation of OxyR is not required for repressing *fimA* transcription regulation, suggesting a constitutive repression of *fimA* expression by OxyR. It is reported that in *E. coli*, expression of Antigen 43, a self-recognizing surface adhesin, is repressed by OxyR (Haagmans & van der Woude, 2000). Similar to our observation in *P. gingivalis*, expression regulation of Ag43 is independent of the oxidation state of OxyR (Wallecha *et al.*, 2003).

*Porphyromonas gingivalis fimA* is present in a single copy in the chromosome (Dickinson *et al.*, 1988). A recent study has shown the presence of monocistronic and polycistronic mRNA of *fimA* (Park *et al.*, 2007). Although the full extent of polycistronic mRNA of *fimA* has not been determined, it contains, at least, the *fimA* gene (pg2132) and its two immediately upstream genes (*pg2130* and *pg2131*). It appears that *fimA* transcription involves a promoter upstream of *pg2130* and an internal promoter immediately upstream of *fimA*. FimR, a transcriptional activator of *fimA*, can bind to promoter upstream of pg2130, but not to the *fimA* promoter region (Nishikawa *et al.*, 2004). Therefore, a cascade regulation of *fimA* expression by FimR is proposed. Activation of *fimA* expression by FimR is through PG2130. We show here a novel regulatory mechanism of *fimA* expression involving OxyR. Unlike FimR, OxyR recognizes a different DNA motif in the internal promoter immediately upstream of *fimA*. The results reveal a complex regulatory system in expression of *P. gingivalis fimA*. Although several adhesive molecules are identified in *P. gingivalis* (Lamont & Jenkinson, 2000), FimA plays an important role in *P. gingivalis* colonization. Further understanding of regulation of FimA production may present attractive targets for inhibition of *P. gingivalis* biofilm formation.

## Statement

OnlineOpen articles are made available in accordance with the terms of the Creative Commons Deed, Attribution 2.5 (further details available from http://www.creativecommons.org), which allows Open Access dissemination of the article, but does not permit commercial exploitation or the creation of derivative works.

## Supplementary material

Table S1 Semi-quantitation RT-PCR products in Fig. 1.

This material is available as part of the online article from: http://www.blackwell-synergy.com/doi/abs/10.1111/j.1574-6968.2008.01116.x (This link will take you to the article abstract).
