# Supplementary material for: OxyR is involved in coordinate regulation of expression of fimA and sod genes in Porphyromonas gingivalis
Source: FEMS Microbiol Lett. 2008 Mar 18;282(2):188–95. doi: 10.1111/j.1574-6968.2008.01116.x (PMC2430334; doi:10.1111/j.1574-6968.2008.01116.x)
Supplement: Table S1 — Semi-quantitation RT-PCR products in Fig. 1. [file fml0282-0188-SD1.doc]

**Supplementary material**

**Table S1.** Semi-quantitation RT-PCR products in Fig. 1.

| 33277/genes | Anaerobic condition | air | Anaerobic condition | air | Anaerobic condition | air |
| --- | --- | --- | --- | --- | --- | --- |
|  | 3 hr | | 6h | | 24 hr | |
| *fimA* | 100.0 | 62.0 | 79.8 | 31.9 | 65.8 | 10.9 |
| *SOD* | 100.0 | 231.8 | 105.0 | 225.8 | 82.4 | 115.2 |
| *pg1737* | 100.0 | 109.5 | 110.5 | 90.3 | 110.2 | 106.6 |

Table S2. Semi-quantitatiion RT-PCR products in Fig. 2.

| genes | 33277 in chamber | 33277 in air | OxyRE in chamber | OxyRE in air |
| --- | --- | --- | --- | --- |
| *fimA* | 100.0 | 43.1 | 140.2 | 118.0 |
| *SOD* | 100.0 | 162.6 | 101.9 | 114.6 |
| *pg1737* | 100.0 | 110.0 | 94.0 | 109.4 |

The PCR products were analyzed by electrophoresis on 1.2% agarose gels, and the bands were photographed. Densitometric analysis was performed by scanning the bands and performing densitometry with AlphaImager 2000 Image Software (Alpha Innotech Corporation). Results are expressed for PCR product as the ratio relative to those of *P. gingivalis* 33277 grown in an anaerobic chamber.

Fig. 1S. Western blot analysis of OxyR production in *P. gingivalis* 33277. P. gingivalis cells were lysed in loading buffer. The proteins were separated by 12% sodium dodecyl sulfate-polyacrylamide gel (SDS-PAGE) and were transferred to nitrocellulose membranes (Gibco BRL) with Mini Transblot Electrophoretic transfer cell (Bio-Rad Laboratories) at 100 V for 1 h. The membrane was treated with 30 ml of blocking solution (3% BSA in PBS containing 0.1% Tween-20, pH 7.4) for 1hr and incubated for 1hr with a polyclonal anti-FimA or anti-Mfa1 antibody diluted 1:1000 in PBS containing 0.1% Tween-20, pH 7.4. The membrane was then rinsed twice and washed three times for 15 min each with 0.1% Tween-20 in PBS. The membrane was incubated with anti-rabbit horseradish peroxidase-conjugated secondary antibodies for 1 hr and rinsed and washed as described above. Antigen-antibody reactivity was visualized by enhanced chemiluminescence (GE Healthcare Bio-Sciences Corp., Piscataway, NJ).

Fig. S1.


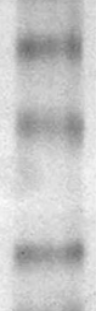

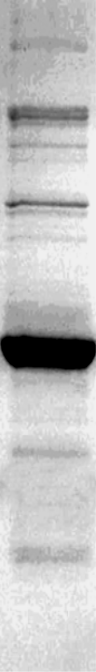


48 KDa

37 KDa

rOxyR
